# Faecal Microbiota of Cats with Insulin-Treated Diabetes Mellitus

**DOI:** 10.1371/journal.pone.0108729

**Published:** 2014-10-03

**Authors:** Erin T. Bell, Jan S. Suchodolski, Anitha Isaiah, Linda M. Fleeman, Audrey K. Cook, Jörg M. Steiner, Caroline S. Mansfield

**Affiliations:** 1 Translational Research and Animal Clinical Trial Study (TRACTS) Group, Faculty of Veterinary Science, The University of Melbourne, Melbourne, Victoria, Australia; 2 Gastrointestinal Laboratory, College of Veterinary Medicine and Biomedical Science, Texas A&M University, College Station, Texas, United States of America; 3 Animal Diabetes Australia, Melbourne, Victoria, Australia; 4 Department of Small Animal Clinical Sciences, College of Veterinary Medicine and Biomedical Science, Texas A&M University, College Station, Texas, United States of America; Charité-University Medicine Berlin, Germany

## Abstract

Microorganisms within the gastrointestinal tract significantly influence metabolic processes within their mammalian host, and recently several groups have sought to characterise the gastrointestinal microbiota of individuals affected by metabolic disease. Differences in the composition of the gastrointestinal microbiota have been reported in mouse models of type 2 diabetes mellitus, as well as in human patients. Diabetes mellitus in cats has many similarities to type 2 diabetes in humans. No studies of the gastrointestinal microbiota of diabetic cats have been previously published. The objectives of this study were to compare the composition of the faecal microbiota of diabetic and non-diabetic cats, and secondarily to determine if host signalment and dietary factors influence the composition of the faecal microbiota in cats. Faecal samples were collected from insulin-treated diabetic and non-diabetic cats, and Illumina sequencing of the 16S rRNA gene and quantitative PCR were performed on each sample. ANOSIM based on the unweighted UniFrac distance metric identified no difference in the composition of the faecal microbiota between diabetic and non-diabetic cats, and no significant differences in the proportions of dominant bacteria by phylum, class, order, family or genus as determined by 16S rRNA gene sequencing were identified between diabetic and non-diabetic cats. qPCR identified a decrease in *Faecalibacterium* spp. in cats aged over ten years. Cat breed or gender, dietary carbohydrate, protein or fat content, and dietary formulation (wet versus dry food) did not affect the composition of the faecal microbiota. In conclusion, the composition of the faecal microbiota was not altered by the presence of diabetes mellitus in cats. Additional studies that compare the functional products of the microbiota in diabetic and non-diabetic cats are warranted to further investigate the potential impact of the gastrointestinal microbiota on metabolic diseases such as diabetes mellitus in cats.

## Introduction

The presence of microorganisms within the mammalian gastrointestinal tract has important consequences for the host, both immunologic and metabolic. Immunologic effects have been recently reviewed [1]. Metabolic effects are largely due to the ability of microorganisms to utilise dietary components that are not digested in the small intestine, such as complex carbohydrates, which are fermented by colonic bacteria to generate short-chain fatty acids such as butyrate, propionate and acetate. These products represent a significant energy source for the host (contributing up to 10% of daily energy requirements) [2], [3], which would otherwise not be available. The gastrointestinal microbiota is also involved in the metabolism of peptides [4], proteins [4] and bile acids [5], the synthesis of bioactive isomers of conjugated linoleic acid that have anti-diabetogenic, anti-obesogenic and anti-atherogenic properties [6], [7], and the regulation of intestinal angiogenesis, epithelial cell proliferation and differentiation [8], [9]. There is significant variation in the composition of gastrointestinal microbiota between individual animals at the bacterial species and strain level [10]–[12]. However, despite this variation the metabolic effects of the microbiota are maintained, suggesting a functional overlap between resident microorganisms.

In acknowledgement of this influence on host metabolism, a potential role for the microbiota in the pathogenesis of metabolic disease has been proposed. Alterations in the composition or functional properties of the microbiota could potentially affect the efficiency of energy acquisition from the diet, intestinal permeability or other metabolic processes within the host, which could in turn influence an individual's susceptibility to metabolic diseases such as obesity and type 2 diabetes mellitus.

In the last decade, a number of studies have reported compositional alterations in the microbiota of obese mice compared with lean mice, with a higher proportion of organisms from the Firmicutes phylum and a corresponding decrease in organisms from the Bacteroidetes phylum associated with an obese phenotype [13]–[15]. This observation is common to both genetic and diet-induced models of obesity, and has also been shown to be reversible with weight loss [14]. Similarly, obesity in humans has been associated with an increased proportion of Firmicutes and a decreased proportion of Bacteroidetes [16], [17]. Weight loss, achieved by either diet or bariatric surgery, was inversely correlated with the proportion of Bacteroidetes in two studies [16], [17]. However, a proportional shift in the opposite direction (i.e. an increase in the ratio of Bacteroidetes to Firmicutes) has also been reported in obese humans [18], as has no difference in the relative proportions of these phyla [19]. In this latter study, although the proportions of Firmicutes and Bacteroidetes were not different between obese and lean people, faecal short chain fatty acid concentration was significantly higher in the obese group. This observation indicates that there may be functional differences in the microbiome associated with obesity, and that these differences can occur independently of compositional differences.

The composition of the microbiota of mice with type 2 diabetes mellitus is also reported to be altered, with an increase in the ratio of Bacteroidetes to Firmicutes being associated with this disease in a mouse model of type 2 diabetes mellitus without obesity [20]. Similar differences in microbiota composition of humans with type 2 diabetes mellitus have been identified [21], [22], with a reduced proportion of Firmicutes and a positive correlation between the ratio of Bacteroidetes to Firmicutes and plasma glucose concentration described in one study [22].

Diabetes mellitus is a common endocrinopathy in cats, with an estimated incidence of 0.5% in first opinion veterinary practice [23]. There are two pathophysiological components of feline diabetes mellitus: (i) reduced insulin secretion from dysfunctional and/or lost pancreatic beta cells, and (ii) insulin resistance, making this disease analogous to type 2 diabetes mellitus in humans [24]. No studies of the gastrointestinal microbiota of diabetic cats have previously been published.

The aims of this study were to compare the faecal microbiota composition of diabetic and non-diabetic cats, and secondarily to determine if host signalment and dietary factors influence the composition of the faecal microbiota in cats.

## Materials and Methods

### Ethics Statement

This study was approved by the University of Melbourne Animal Ethics Committee, using National Health and Medical Research Council (NHMRC) guidelines.

### Animals and Sample Collection

All cats involved in this study were owned, pet cats. Cats were diagnosed with diabetes mellitus on the basis of appropriate clinical signs (polyuria, polydipsia, polyphagia and weight loss) and clinical pathology findings (persistent hyperglycaemia and glucosuria). Both newly diagnosed and long-term diabetic cats were considered for inclusion in the study. All diabetic cats received exogenous insulin as one component of their therapy. Non-diabetic cats were clinically healthy and had not been previously diagnosed with diabetes mellitus. Non-diabetic cats were breed-, age- (within three years) and sex-matched to diabetic cats.

Naturally voided faecal samples were collected from the diabetic and non-diabetic cats at home or at a veterinary clinic. Samples were refrigerated at 4°C until transport to the laboratory, which was completed within 48 hours of sample collection. Samples were then frozen at -20°C until processing.

### Sequencing of 16S rRNA genes

An aliquot of 100 mg (wet weight) of each faecal sample was extracted by a bead-beating method using a commercial DNA extraction kit (ZR Fecal DNA Kit, Zymo Research Corporation) following the manufacturer's instructions. The bead beating step was performed on a homogenizer (FastPrep-24, MP Biomedicals) for 60 seconds at a speed of 4 metres per second.

The V4 region of the 16S rRNA gene was amplified with primers 515F (5′-GTGCCAGCMGCCGCGGTAA-3′) and 806R (5′-GGACTACVSGGGTATCTAAT-3′) at the MR DNA Laboratory (Shallowater, TX, USA). A 100 ng (1 µl) aliquot of each DNA sample was used for a 50 µl PCR reaction. HotStarTaq Plus Master Mix Kit (Qiagen, Valencia, CA, USA) was used for PCR under the following conditions: 94°C for 3 min followed by 32 cycles of 94°C for 30 sec; 60°C for 40 sec and 72°C for 1 min; and a final elongation step at 72°C for 5 min. PCR amplification products were verified on 2% agarose gels and samples were purified using calibrated Ampure XP beads (Agencourt Bioscience Corporation, Danvers, MA, USA). The Nextera DNA sample Preparation kit including sequencing adapters and sample specific barcodes was used to prepare a DNA library and sequenced at MR DNA on an Illumina MiSeq instrument.

### Quantitative PCR (qPCR)

To evaluate bacterial genera that are typically present at very low abundance or not detected in sequence data based on our experience from previous studies [12], [25] we performed qPCR assays for selected bacterial groups: total bacteria, *Lactobacillus* spp., *Bifidobacterium* spp. and *Faecalibacterium* spp.. The oligonucleotide sequence of primers and respective annealing temperatures are summarised in Table 1. The DNA concentration of all faecal samples was adjusted to 5 ng µL^−1^. A commercial real-time PCR thermocycler (CFX96™, Biorad Laboratories, Hercules, CA, USA) was used for all experiments. Standard curves using 1∶10 dilutions of DNA (ranging from 2 ng to 0.2 pg) from lyophilized bacterial species of each genus (Faecalibacterium prausnitzii (ATCC 27766); Lactobacillus rhamnosus GG (ATCC 53103); Bifidobacterium bifidum (ATCC 11863)) and feline fecal community DNA for universal bacteria were used to calculate the unknown bacterial genomic targets. All samples and standards were run in duplicate. SYBR-based reaction mixtures (total 10 µL) contained 5 µL of SsoFastTM EvaGreen supermix (Biorad Laboratories, Hercules, CA, USA), 2.6 µL of water, 0.4 µL of each primer (final concentration: 400 nM), and 2 µL of DNA (1∶10 or 1∶100 dilution). PCR conditions were 95°C for 2 min, and 40 cycles at 95°C for 5 sec and 10 sec at the optimized annealing temperature. After all PCR cycles were completed, a melt curve analysis was performed for SYBR-based qPCR assays under the following conditions: 1 min at 95°C, 1 min at 55°C, and 80 cycles of 0.5°C increments (10 sec each). The qPCR data was expressed as log amount of DNA (fg) for each particular bacterial group per 10 ng of isolated total DNA.

**Table 1:** Oligonucleotide sequences of primers and annealing temperatures used for this study.

qPCR primers	Sequence (5′-3′)	Target	Annealing (°C)	Reference
BifF	TCGCGTCYGGTGTGAAAG	Bifidobacterium	60	[47]
BifR	CCACATCCAGCRTCCAC			
FaecaF	GAAGGCGGCCTACTGGGCAC	*Faecalibacterium*	60	[48]
FaecaR	GTGCAGGCGAGTTGCAGCCT			
LactF	AGCAGTAGGGAATCTTCCA	*Lactobacillus*	58	[47]
LactR	CACCGCTACACATGGAG			
341-F	CCTACGGGAGGCAGCAGT	Universal bacteria	59	[46]
518-R	ATTACCGCGGCTGCTGG			

### Statistical analysis of sequencing data

The raw sequence data were demultiplexed by barcodes, and low quality reads were filtered using the QIIME v1.8 (http://qiime.sourceforge.net) database's default parameters [26]. A total of 1,078,487 (median: 35,437; range 22,511–53,163 sequences per sample) were obtained. For further analysis, each sample was rarefied to an even sequencing depth of 22,500 sequences per sample to adjust for uneven sequencing depth across all samples. Sequences were then clustered into operational taxonomic units (OTUs) using a closed-reference OTU picking protocol at the 97% sequencing identity level using UCLUST [27] against the Greengenes database, pre-clustered at 97% sequence identity [28], [29]. Data was uploaded to the database of the National Centre for Biotechnology Information (NCBI) (accession number SRP043386).

The compiled data were used to determine the relative percentages of bacteria for each individual sample. Alpha rarefaction and beta diversity measures were calculated and plotted using QIIME. Differences in microbial communities between groups were investigated using the phylogeny-based unweighted UniFrac distance metric. This analysis measures the phylogenetic distance among bacterial communities in a phylogenetic tree, and thereby provides a measure of similarity among microbial communities present in different biological samples. The groups considered for analysis were (i) diabetic versus non-diabetic cats; (ii) domestic shorthair cats versus cats of other breeds; (iii) male versus female cats; (iv) cats aged ten years or less versus cats greater than ten years of age; (v) protein content of the diet: moderate (6.0–10.4 grams of protein per 100 kcal metabolisable energy (ME)) versus high (10.5–13.1 grams of protein per 100 kcal ME); (vi) carbohydrate content of the diet: low (2.9–4.9 grams of carbohydrate per 100 kcal ME) versus moderate (5.0–12.5 grams of carbohydrate per 100 kcal ME); (vii) fat content of the diet: low (3.6–4.9 grams of fat per 100 kcal ME) versus moderate (5.0–6.4 grams of fat per 100 kcal ME). Differences in microbial communities between these groups were investigated by visual assessment for clustering using principal coordinates analysis (PCoA) plots, and by analysis of similarity (ANOSIM) calculated on unweighted UniFrac distances using the statistical software package PRIMER 6 (PRIMER-E Ltd, Luton, UK) [30].

Differences in the median ages of diabetic versus non-diabetic cats were examined by two-sided Mann-Whitney U tests (IBM SPSS Statistics, Version 22, IBM Corp., Armonk, NY, USA). Differences in the proportions of bacteria (defined as median percentage of total sequences) by phyla, class, order, family, and genus between diabetic and non-diabetic cats were assessed by two-sided Mann-Whitney U tests (IBM SPSS Statistics, Version 22, IBM Corp., Armonk, NY, USA). Only groups present in at least 50% of cats were included in this analysis. The ratio of Bacteroidetes to Firmicutes in each cat was calculated and a linear regression model was used to assess for an association between this ratio and the presence of diabetes mellitus. *P* values <0.05 were considered statistically significant.

### Statistical analysis of qPCR data

The mean counts of each bacterial group in diabetic versus non-diabetic cats, and cats aged ten years or less versus cats greater than ten years old, were compared by 2-sample t tests (IBM SPSS Statistics, Version 22, IBM Corp, Armonk, New York, USA). *P* values <0.05 were considered statistically significant.

## Results

### Study population

A total of 30 (ten diabetic and 20 non-diabetic) cats were enrolled into the study. Signalment and dietary information of these cats is summarised in Table 2. Ten of the non-diabetic cats (cats 11–20) were breed, age- and sex-matched to diabetic cats, and these cats formed the control group for comparison of the microbiota between diabetic and non-diabetic cats. The remaining ten non-diabetic cats (cats 21–30) were included in analysis of the effects of signalment and dietary factors on microbiota composition.

**Table 2:** Signalment and dietary information for enrolled cats.

Cat ID	Diabetic (yes or no)	Age (years)	Breed	Sex	Dietary protein content	Dietary carbohydrate content	Dietary fat content
**1**	Yes	11	DSH	Female	High	Low	Moderate
**2**	Yes	13	Burmese	Male	High	Low	Low
**3**	Yes	12	Burmese	Male	High	Low	Low
**4**	Yes	14	DSH	Male	Moderate	Moderate	Moderate
**5**	Yes	4	DSH	Male	High	Low	Low
**6**	Yes	12	DSH	Male	High	Low	Low
**7**	Yes	8	Burmese	Male	Moderate	Low	Moderate
**8**	Yes	18	DSH	Female	Moderate	Low	Moderate
**9**	Yes	9	DSH	Female	Moderate	Low	Moderate
**10**	Yes	12	Siamese	Female	High	Low	Moderate
**11**	No^#^	5	DSH	Female	Moderate	Moderate	Moderate
**12**	No^#^	14	DSH	Male	Moderate	Moderate	Low
**13**	No^#^	16	Burmese	Male	Moderate	Moderate	Moderate
**14**	No^#^	14	Siamese	Female	Moderate	Moderate	Moderate
**15**	No^#^	11	Burmese	Male	Moderate	Moderate	Low
**16**	No^#^	15	DSH	Male	Moderate	Moderate	Moderate
**17**	No^#^	11	DSH	Female	Not known	Not known	Not known
**18**	No^#^	15	DSH	Female	Moderate	Low	Moderate
**19**	No^#^	9	DSH	Female	Moderate	Moderate	Low
**20**	No^#^	8	Burmese	Male	High	Normal	Low
**21**	No	6	Burmese	Male	Moderate	Moderate	Low
**22**	No	2	DSH	Male	High	Low	Low
**23**	No	2	DSH	Male	Moderate	Moderate	Moderate
**24**	No	5	DSH	Female	High	Low	Low
**25**	No	14	DSH	Female	Moderate	Moderate	Moderate
**26**	No	6	DSH	Male	Moderate	Moderate	Moderate
**27**	No	5	DSH	Female	High	Low	Low
**28**	No	5	DSH	Female	Moderate	Moderate	Moderate
**29**	No	16	DSH	Male	Moderate	Moderate	Low
**30**	No	6	DSH	Male	High	Low	Low

DSH  =  Domestic Shorthair. Dietary protein content: moderate 6.0–10.4 g/100 kcal metabolisable energy (ME); high 10.5–13.1 g/100 kcal ME. Dietary carbohydrate content: low 2.9–4.9 g/100 kcal ME; moderate 5.0–12.5 g/100 kcal ME. Dietary fat content: low 3.6–4.9 g/100 kcal ME; moderate 5.0–6.4 g/100 kcal ME. ^#^ denotes inclusion in the non-diabetic control group for comparison of the microbiota between diabetic and non-diabetic cats.

### Composition of faecal microbiota as determined by sequencing of the 16S rRNA gene

**Figure 1:**
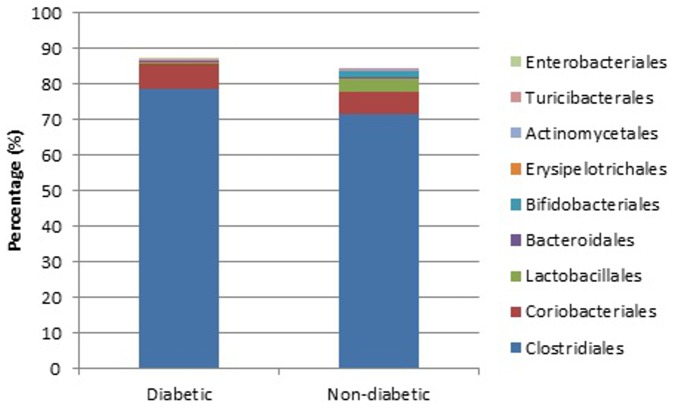
Median percentage of bacterial orders identified in diabetic and non-diabetic cats.

The predominant bacterial phyla in all cats were Firmicutes, Actinobacteria and Bacteroidetes; together these phyla comprised on average greater than 98% of the total bacterial sequences (mean 98.29%, standard deviation (SD) 3.66%). The predominant bacterial orders in diabetic and non-diabetic cats are shown in Figure 1. Table 3 summarises the proportions of bacteria by phyla, class, order, family, and genus in diabetic and non-diabetic cats. There was no significant difference in the relative proportions of any of these taxa between diabetic and non-diabetic cats. The ratio of Bacteroidetes to Firmicutes was not significantly associated with the presence of diabetes mellitus (*P* = 0.174).

**Table 3:** Relative proportions of predominant bacterial taxa identified by sequencing of the 16S rRNA gene.

	Median percentage of sequences		
	Diabetic cats (minimum-maximum)	Non-diabetic cats (minimum-maximum)	P-value
**PHYLUM**			
Actinobacteria	8.79 (1.60–38.42)	9.90 (3.82–34.94)	0.273
Bacteroidetes	0.15 (0.06–2.62)	0.47 (0.11–3.85)	0.061
Euryarchaeota	0.01 (0.00–14.13)	0.01 (0.00–0.02)	0.393
Firmicutes	83.79 (59.82–97.68)	89.44 (64.85–95.18)	0.470
Fusobacteria	0.02 (0.00–1.51)	0.01 (0.00–0.19)	0.381
Proteobacteria	0.18 (0.06–9.64)	0.17 (0.07–1.05)	0.470
Tenericutes	0.04 (0.02–0.16)	0.04 (0.01–0.08)	0.912
**CLASS**			
Actinobacteria (class)	0.29 (0.04–38.24)	1.78 (0.08–33.94)	0.406
Bacilli	1.89 (0.16–51.59)	3.44 (0.20–41.43)	0.650
Bacteroidia	0.15 (0.06–2.62)	0.47 (0.11–3.85)	0.121
Betaproteobacteria	0.01 (0.00–1.51)	0.01 (0.00–0.02)	0.821
Clostridia	78.79 (8.20–96.92)	71.49 (31.64–94.15)	0.406
Coriobacteria	6.88 (0.18–16.38)	6.49 (1.00–21.37)	0.880
Deltaproteobacteria	0.04 (0.01–0.19)	0.04 (0.02–0.23)	0.521
Erysipelotrichi	0.13 (0.04–6.28)	0.19 (0.01–23.40)	0.623
Fusobacteria	0.02 (0.00–1.51)	0.01 (0.00–0.19)	0.762
Gammaproteobacteria	0.08 (0.03–9.59)	0.07 (0.03–0.75)	0.821
Methanobacteria	0.01 (0.00–14.09)	0.00 (0.00–0.00)	0.241
**ORDER**			
Actinomycetales	0.13 (0.02–38.15)	0.07 (0.03–0.49)	0.597
Bacillales	0.03 (0.01–42.42)	0.03 (0.00–1.99)	0.970
Bacteroidales	0.15 (0.06–2.62)	0.47 (0.11–3.85)	0.121
Bifidobacteriales	0.08 (0.12–8.72)	1.68 (0.04–33.91)	0.096
Burkholderiales	0.01 (0.00–1.51)	0.01 (0.00–0.02)	0.821
Clostridiales	78.79 (8.20–96.92)	71.49 (31.64–94.15)	0.406
Coriobacteriales	6.88 (0.18–16.38)	6.49 (1.00–21.37)	0.880
Enterobacteriales	0.08 (0.03–9.59)	0.04 (0.02 –0.75)	0.307
Erysipelotrichales	0.13 (0.04–6.28)	0.19 (0.01–23.40)	0.623
Fusobacteriales	0.02 (0.00–1.51)	0.01 (0.00–0.19)	0.762
Lactobacillales	0.53 (0.12–21.2)	3.37 (0.14–40.89)	0.364
Methanobacteriales	0.01 (0.00–14.09)	0.00 (0.00–0.01)	0.241
Turicibacterales	0.09 (0.02–15.60)	0.10 (0.04–4.47)	0.940
**FAMILY**			
Actinomycetaceae	0.08 (0.00–0.33)	0.04 (0.00–0.26)	0.684
Alcaligenaceae	0.01 (0.00–1.51)	0.01 (0.00–0.02)	0.853
Bacteroidaceae	0.05 (0.03–2.40)	0.24 (0.04–3.69)	0.089
Bifidobacteriaceae	0.08 (0.02–8.72)	1.68 (0.04–33.91)	0.105
Carnobacteriaceae	0.01 (0.00–6.08)	0.01 (0.00–0.05)	0.190
Clostridiaceae	22.96 (2.70–38.04)	22.79 (1.75–41.30)	0.796
Clostridiaceae unclassified	11.33 (0.40–20.20)	10.88 (4.84–14.32)	0.739
Coriobacteriaceae	6.88 (1.18–16.38)	6.49 (1.00–21.37)	0.912
Corynebacteriaceae	0.02 (0.00–0.07)	0.02 (0.00–0.32)	0.579
Desulfovibrionaceae	0.04 (0.01–0.19)	0.04 (0.02–0.23)	0.529
Enterobacteriaceae	0.08 (0.03–9.59)	0.04 (0.02–0.75)	0.315
Enterococcaceae	0.32 (0.05–2.26)	0.58 (0.06–40.59)	0.631
Erysipelotrichaceae	0.13 (0.04–6.28)	0.19 (0.01–23.40)	0.631
Eubacteriaceae	0.02 (0.00–0.47)	0.02 (0.00–5.82)	0.436
Fusobacteriaceae	0.02 (0.00–1.51)	0.01 (0.00–0.19)	0.796
Lachnospiraceae	36.35 (0.73–54.23)	20.38 (9.03–63.18)	0.143
Lactobacillaceae	0.08 (0.03–0.12)	0.08 (0.03–32.52)	0.971
Methanobacteriaceae	0.01 (0.00–0.14)	0.00 (0.00–0.01)	0.247
Micrococcaceae	0.02 (0.00–35.67)	0.01 (0.00–0.04)	0.481
Mogibacteriaceae	0.04 (0.00–0.33)	0.04 (0.00–8.18)	0.529
Peptococcaceae	0.06 (0.01–4.69)	2.07 (0.03–9.64)	0.089
Peptostreptococcaceae	2.10 (0.17–15.67)	1.76 (0.16–21.31)	0.853
Planococcaceae	0.00 (0.00–12.31)	0.01 (0.00–0.18)	0.579
Porphyromonadaceae	0.00 (0.00–0.19)	0.02 (0.00–0.30)	0.143
Ruminococcaceae	1.54 (0.00–9.21)	1.55 (0.00–12.01)	0.684
Staphylococcaceae	0.03 (0.01–28.60)	0.02 (0.00–1.72)	0.971
Streptococcaceae	0.04 (0.01–20.99)	0.05 (0.01–9.30)	0.796
Turicibacteraceae	0.09 (0.02–15.60)	0.10 (0.04–4.47)	0.971
**GENUS**			
Actinomyces	0.08 (0.00–0.33)	0.04 (0.00–0.24)	0.684
Anaerofustis	0.02 (0.00–0.46)	0.00 (0.00–0.28)	0.218
Arthrobacter	0.02 (0.00–35.66)	0.01 (0.00–0.04)	0.631
Bacteroides	0.05 (0.03–2.40)	0.24 (0.04–3.69)	0.089
Bifidobacterium	0.02 (0.00–6.15)	0.03 (0.01–33.55)	0.105
Bifidobacterium unclassified	0.06 (0.00–2.57)	0.88 (0.02–19.76)	0.105
Blautia	12.44 (0.16–19.60)	9.61 (2.68–28.83)	0.739
Candidatus Arthromitus	0.00 (0.00–0.02)	0.00 (0.00–1.76)	0.796
Carnobacterium	0.01 (0.00–5.22)	0.01 (0.00–0.04)	0.684
Catenibacterium	0.01 (0.00–0.07)	0.02 (0.00–21.76)	0.315
Clostridium	7.15 (0.95–22.55)	3.97 (0.13–13.53)	0.247
Clostridium unclassified 1	11.33 (0.40–20.20)	10.88 (4.84–14.32)	0.739
Clostridium unclassified 2	13.34 (0.31–28.11)	14.60 (0.56–32.82)	0.971
Collinsella	5.52 (0.12–15.02)	5.91 (0.40–20.15)	0.796
Coprococcus	0.49 (0.02–14.87)	1.08 (0.13–5.17)	0.190
Coriobacterium unclassified	0.06 (0.00–1.39)	0.28 (0.02–1.03)	0.218
Corynebacterium	0.02 (0.00–0.07)	0.02 (0.00–0.32)	0.579
Dorea	3.25 (0.04–9.46)	2.55 (0.46–7.29)	0.529
Enterobacteriacium unclassified	0.08 (0.03–9.59)	0.04 (0.02–0.75)	0.353
Enterococcus	0.32 (0.05–2.26)	0.58 (0.06–40.59)	0.631
Epulopiscium	0.01 (0.00–1.23)	0.01 (0.00–0.95)	0.579
Erysipelothrix unclassified	0.00 (0.00–1.52)	0.00 (0.00–0.18)	0.739
Eubacterium	0.03 (0.01–6.20)	0.06 (0.00–1.79)	0.393
Fusobacterium	0.02 (0.00–1.51)	0.01 (0.00–0.19)	0.796
Lachnospira unclassified	13.15 (0.19–36.15)	6.63 (2.24–23.55)	0.218
Lactobacillus	0.07 (0.03–0.11)	0.06 (0.02–14.06)	0.739
Lactococcus	0.01 (0.00–0.10)	0.01 (0.00–9.16)	0.739
Methanobrevibacter	0.01 (0.00–0.12)	0.00 (0.00–0.01)	0.315
Mogibacterium unclassified	0.04 (0.01–0.33)	0.04 (0.00–8.18)	0.529
Oscillospira	0.19 (0.13–0.45)	0.25 (0.16–1.00)	0.280
Parabacteroides	0.00 (0.00–0.19)	0.02 (0.00–0.30)	0.143
Pediococcus	0.01 (0.00–0.02)	0.01 (0.00–18.47)	0.393
Peptococcus	0.05 (0.01–4.69)	2.07 (0.02–9.63)	0.105
Peptostreptococcus unclassified	2.07 (0.16–15.65)	1.66 (0.05–21.17)	0.796
Pseudoramibacter Eubacterium	0.00 (0.00–0.01)	0.00 (0.00–5.82)	0.393
Roseburia	0.02 (0.00–0.29)	0.11 (0.01–0.42)	0.247
Ruminococcus 1	0.25 (0.02–5.80)	0.80 (0.16–2.35)	0.190
Ruminococcus 2	0.08 (0.02–0.32)	0.07 (0.04–1.07)	0.684
Ruminococcus unclassified	1.28 (0.11–8.55)	1.18 (0.48–11.13)	0.853
Slackia	0.20 (0.04–1.34)	0.44 (0.03–1.02)	0.796
SMB53	0.09 (0.00–0.37)	0.07 (0.00–0.18)	0.796
Sporosarcina	0.00 (0.00–10.50)	0.01 (0.00–0.16)	0.684
Staphylococcus	0.03 (0.01–28.60)	0.02 (0.00–1.72)	0.971
Streptococcus	0.03 (0.01–20.97)	0.02 (0.00–3.49)	0.579
Turicibacter	0.09 (0.02–15.60)	0.10 (0.04–4.47)	0.971

Differences in median percentages between diabetic and non-diabetic cats were calculated using Mann-Whitney U tests. P values <0.05 were considered significant.

**Figure 2:**
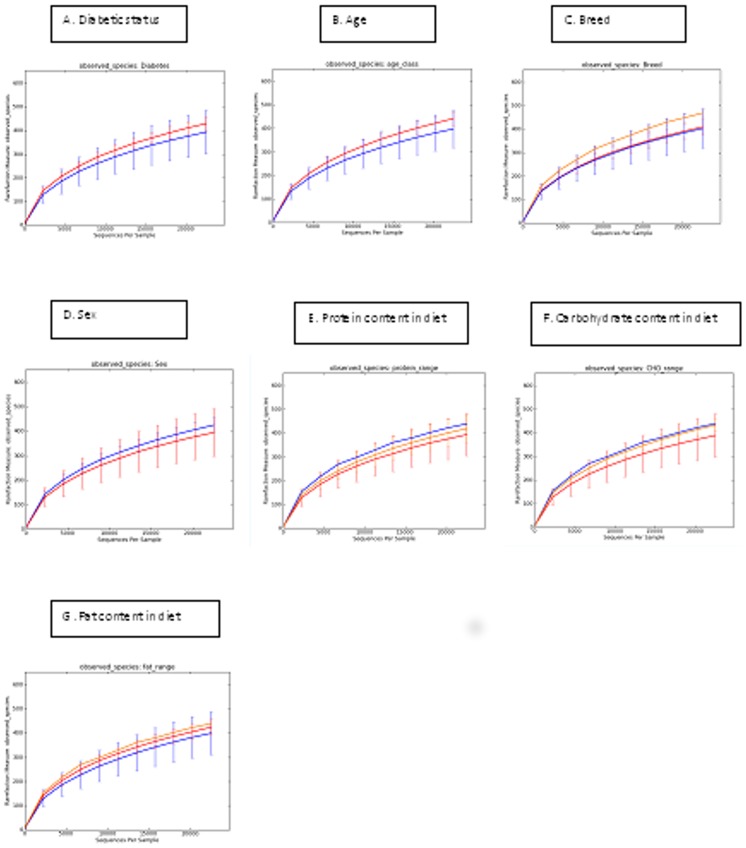
Rarefaction analysis of 16S rRNA gene sequences obtained from faecal samples divided into diabetic, signalment and dietary groups. A: Diabetic status (blue: diabetic, red: non-diabetic); B: Age (blue: cats greater than ten years of age, red: cats aged ten years or less); C: Breed (blue: DSH, red: Burmese, yellow: Siamese); D: Sex (blue: male, red: female); E: Protein content of diet (blue: N/A, red: high (10.5–13.1 grams of protein per 100 kcal metabolisable energy (ME)), yellow: moderate (6.0–10.4 grams of protein per 100 kcal ME)); F: Carbohydrate content of diet (blue: N/A, red: low (2.9–4.9 grams of carbohydrate per 100 kcal ME), yellow: moderate (5.0–12.5 grams of carbohydrate per 100 kcal ME)); G: Fat content of diet (blue: moderate (5.0–6.4 grams of fat per 100 kcal ME), red: low (3.6–4.9 grams of fat per 100 kcal ME), yellow: N/A).

Rarefaction analysis was performed at a uniform depth of 22,500 sequences per sample. No significant differences in alpha diversity were observed for any of the evaluated parameters (Figure 2).

**Figure 3:**
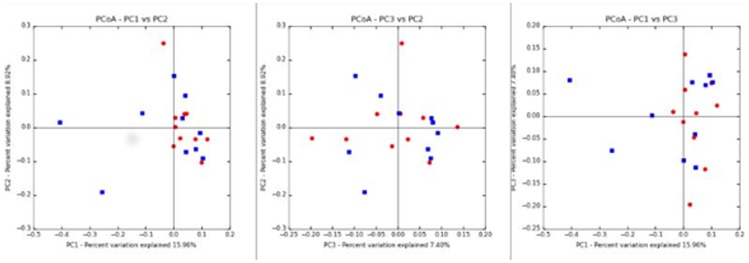
Principal coordinates analysis (PCoA) of unweighted UniFrac distances of 16S rRNA. Blue: diabetic cat, red: non-diabetic cat.

**Figure 4:**
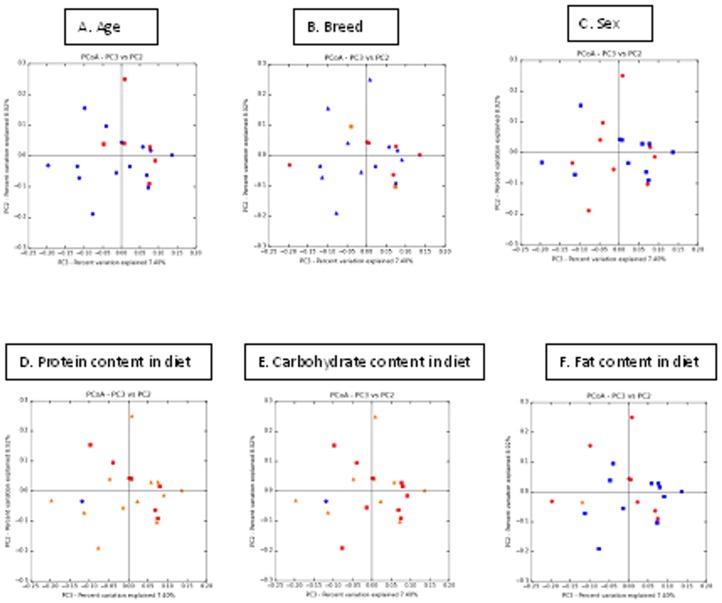
Principal coordinates analysis (PCoA) of unweighted UniFrac distances of 16S rRNA. A: Age (blue: cats greater than ten years of age, red: cats aged ten years or less); B: Breed (blue: DSH, red: Burmese, yellow: Siamese); C: Sex (blue: male, red: female); D: Protein content of diet (blue: N/A, red: high (10.5–13.1 grams of protein per 100 kcal metabolisable energy (ME)), yellow: moderate (6.0–10.4 grams of protein per 100 kcal ME)); E: Carbohydrate content of diet (blue: N/A, red: low (2.9–4.9 grams of carbohydrate per 100 kcal ME), yellow: moderate (5.0–12.5 grams of carbohydrate per 100 kcal ME)); F: Fat content of diet (blue: moderate (5.0–6.4 grams of fat per 100 kcal ME), red: low (3.6–4.9 grams of fat per 100 kcal ME), yellow: N/A).

Principal coordinates analysis plots based on the unweighted UniFrac distance metric are shown in Figure 3 (diabetic versus non-diabetic cats) and Figure 4. ANOSIM calculated on the unweighted UniFrac distance metric identified no significant differences in the UniFrac distances between diabetic and non-diabetic cats (*P* = 0.84), or between any of the other signalment or dietary factors considered (Table 4).

**Table 4:** Summary of ANOSIM results for the factors evaluated in this study.

Variable	R statistic	p-value
Diabetes mellitus	−0.0496	0.84
Age	0.1339	0.11
Breed	0.0184	0.42
Sex	−0.0455	0.76
Dietary carbohydrate	−0.0338	0.68
Dietary fat	0.025	0.36
Dietary protein	−0.011	0.45

ANOSIM was calculated using unweighted UniFrac distances. P values <0.05 were considered significant.

### qPCR evaluation of the faecal microbiota

The mean counts of each bacterial group in diabetic and non-diabetic cats are summarised in Table 5. The mean counts of each bacterial group in cats aged ten years or younger and cats aged greater than ten years are summarised in Table 6. *Faecalibacterium* spp. were significantly lower in cats greater than ten years of age (mean ± SD 5.38±0.96) compared with cats ten years of age or younger (mean ± SD 6.39±0.74) (*P* = 0.035). No differences in the mean counts of the other bacterial groups on the basis of diabetes or age were identified.

**Table 5:** Quantitative PCR evaluation of the faecal microbiota in diabetic versus non-diabetic cats.

	Mean amount of bacteria		
	Diabetic cats	Non-diabetic cats	*P*-value
**All bacteria**	11.86±0.10	11.79±0.23	0.443
***Bifidobacterium***	4.06±1.28	5.38±1.75	0.072
***Faecalibacterium***	5.33±1.17	6.04±0.69	0.118
***Lactobacillus***	4.14±0.55	4.33±0.73	0.517

Values are expressed as means ± standard deviation of the log amount of DNA (fg) per 10 ng of isolated total DNA. Differences in mean values between diabetic and non-diabetic cats were determined by 2-sided t-tests. P values <0.05 were considered significant.

**Table 6:** Quantitative PCR evaluation of the faecal microbiota in adult versus geriatric cats.

	Mean amount of bacteria		
	Adult cats	Geriatric cats	*P*-value
**All bacteria**	11.84±0.14	11.82±0.19	0.807
***Bifidobacterium***	5.39±1.70	4.43±1.58	0.241
***Faecalibacterium***	6.39±0.74	5.38±0.96	*0.035*
***Lactobacillus***	4.21±0.82	4.25±0.57	0.894

Values are expressed as means ± standard deviation of the log amount of DNA (fg) per 10 ng of isolated total DNA. Differences in mean values between cats aged ten years or younger (“adult”) and cats greater than ten years (“geriatric”) were determined by 2-sided t-tests. P values <0.05 were considered significant.

## Discussion

This study is the first to describe the faecal microbiota composition of cats with diabetes mellitus, and contributes to existing knowledge of the feline gastrointestinal microbiota. In our study, Firmicutes was the predominant bacterial phylum in both diabetic and non-diabetic cats, and Firmicutes, Actinobacteria and Bacteroidetes together represented on average greater than 98% of total bacteria sequenced in both groups. These results are consistent with those of Handl et al. [12], who used 16S rRNA gene pyrosequencing to describe the faecal microbiota of 12 healthy pet cats. They also reported that greater than 99% of total bacteria identified belonged to the phyla Firmicutes, Actinobacteria and Bacteroidetes, although the percentage contributions by each individual phylum (Firmicutes 92%, Actinobacteria 7.3%, Bacteroidetes 0.45%) differed from that of our study.

In general, there is agreement that Firmicutes, Actinobacteria and Bacteroidetes are dominant bacterial phyla in feline faecal samples [11]. However, descriptions of the feline microbiota vary between studies, likely as determination of the relative abundances of bacteria is influenced by sample population, the sample handling, and also the molecular technique that is employed [25], [31]. Actinobacteria was determined to be the most prevalent bacterial phylum in feline faecal samples when an alternative target gene (the chaperonin (*cpn*60) gene) was amplified for sequencing [10], and when investigated by fluorescent in situ hybridisation [32], [33]. Inter-laboratory differences in DNA extraction, sample handling, and storage protocols are also potential sources of variation between studies [34]. Further confounding interpretation of results is the fact that the composition of the microbiota varies along the gastrointestinal tract, and consequently faecal microbiota may not be representative of the microbiota in the various segments of the gastrointestinal tract [31], [34], [35]. These factors complicate study of the gastrointestinal microbiota, and direct comparison of results between studies may be problematic. However, comparison of the composition of the microbiota between groups of animals within a study such as ours is not subject to as many of these limitations, and is likely to generate more meaningful results.

Our results showed that the presence of insulin-treated diabetes mellitus in cats does not affect faecal microbiota composition, as evaluated by the UniFrac distance metric or by comparison of relative abundances of predominant bacterial taxa identified by sequencing of the 16S rRNA gene. We were therefore unable to replicate the results of Serino et al. [20] who described a decreased proportion of Firmicutes in mice with type 2 diabetes mellitus, or Larsen et al. [22] who reported a similar finding in type 2 diabetic men, in cats with diabetes mellitus. It is possible that the inability to identify a difference in microbiota composition between diabetic and non-diabetic cats could have been due to the relatively small sample size in this study; however, previous studies that have reported compositional differences of the microbiota associated with obesity [16], type 2 diabetes [22] and type 1 diabetes [36] have studied a similar number of or fewer individuals, making type II error unlikely. An additional consideration is that all diabetic cats in this study were treated with insulin, this being standard therapy for feline diabetes mellitus. Whether or not exogenous insulin can alter microbiota composition and/or obscure diabetes-associated changes in microbiota composition is unknown, however future studies could explore this issue by studying diabetic cats at the time of diagnosis, prior to commencement of insulin therapy.

Compositional analysis of the microbiota, as undertaken in this study, may overlook the complexities of microbial communities in vivo. In a recent study, faecal microbiota of children was examined at several time points up to three years of age, and the microbiota composition of children who developed anti-islet cell antibodies (a marker of type 1 diabetes) was compared with children who remained antibody-free [37]. No differences in microbiota composition, relative proportions of bacteria at genus level, or diversity were noted between groups. However when a microbial correlation network was constructed (by determining correlation values between all possible genera pairs), a significant difference was noted in microbial interaction networks between the two groups of children. It was concluded that despite an absence of compositional differences, microbial interaction networks were compromised in children who developed anti-islet cell antibodies. This study demonstrates that disease-associated alterations of the faecal microbiota may not necessarily be discernible as quantitative compositional changes; and that consideration of intra-microbiota relationships may afford a more comprehensive assessment of the microbiota.

Importantly, failure to identify compositional differences of faecal microbiota between diabetic and non-diabetic cats does not exclude the possibility of functional differences of the microbiota in affected individuals. Host metabolic effects may not be entirely predictable by a particular microbiota composition, as there is a large functional overlap in metabolic roles of bacteria within the gastrointestinal tract [38]. A metagenomic analysis of faecal microbiota in people with type 2 diabetes demonstrated that the disease was associated with marked functional alterations of the microbiota but only moderate compositional change [39]. Future studies that employ metagenomic, transcriptomic, or metabolomics approaches could identify functional differences of the microbiota in diabetic cats that are not manifest as an overall difference in microbiota composition.

The composition of the microbiota has been reported to change associated with age in humans, with the most consistent change reported being a decreased total proportion and species diversity of bifidobacteria in elderly people [40]–[42]. In cats, the microbiota composition is more diverse in kittens pre-weaning than post-weaning [33]. Longer term effects have not been comprehensively investigated, although one group reported no difference in bifidobacteria counts of kittens compared with geriatric cats [43]. Specific age-associated differences in the proportions of predominant bacterial taxa or *Bifidobacterium* spp. were not identified in our study, although *Faecalibacterium* spp. were decreased in cats greater than ten years of age. Interestingly, reduced levels of *Faecalibacterium* spp. have also been reported in elderly humans [44], [45]. Further studies that compare samples from very young and very old cats may more readily identify age-related alterations in microbiota composition of cats.

None of the dietary factors that we evaluated affected faecal microbiota composition, in contrast to some previous studies which have related high protein diets to a lower abundance of Bifidobacterium [33], [43], [46]. However, the diets investigated in those studies differed with respect to other nutrients as well as protein, and the effect of individual dietary components in isolation has not been scrutinised. All these previous studies have also utilised laboratory-housed cats, for which dietary and environmental factors can be more tightly controlled than for the pet cats in our study. In our study cats were fed a variety of commercially available diets, many of which were designed to meet maintenance requirements of adult cats. The variability in consumed diets also meant that only small groups of cats were available for comparison for some of the dietary factors considered, which may have impaired our ability to detect diet-associated differences. It is possible that with more extreme differences in nutrient profiles and/or studies involving larger numbers of cats, diet-related alterations in microbiota composition would become apparent. Further studies that are specifically designed to investigate individual nutrient effects are needed to ascertain the significance of diet in influencing microbiota composition in cats.

In conclusion, the faecal microbiota composition of insulin-treated, diabetic cats determined by 16S rRNA gene sequencing did not differ from that of non-diabetic cats in this study. qPCR identified a decrease in *Faecalibacterium* spp. in elderly cats, similar to observations in elderly humans. There were no differences in faecal microbiota composition associated with cat breed or gender, dietary protein, carbohydrate or fat content, or dietary formulation in our study population of pet cats. Additional studies that compare the functional products of the microbiota in diabetic and non-diabetic cats are warranted, to further investigate the potential pathogenetic role of the gastrointestinal microbiota in metabolic diseases such as diabetes mellitus in cats.
